# Eruptive disseminated spitz nevus: a case report and review of the genetic aspect of the disease^⋆^

**DOI:** 10.1016/j.abd.2023.02.010

**Published:** 2023-11-28

**Authors:** Emre Zekey, Seher Darakcı

**Affiliations:** a Department of Dermatology, Sivas Numune Hospital, Sivas, Turkey; b Department of Pathology, Sivas Numune Hospital, Sivas, Turkey

*Dear Editor,*

Spitz nevi are usually solitary, benign proliferations of epithelioid melanocytes. Various clinical and histopathological subtypes of Spitz nevi have been reported in the literature over time. The eruptive variant of Spitz nevus has been reported in 34 cases so far. Genetic analysis was performed in 7 of them. In this report, the authors present a patient with hundreds of Spitz nevi in her body in just 2 months and review the current literature together with the genetic studies.

A 17-year-old young woman presented with multiple nevi starting from the buttocks and spreading to the back and legs for the last 2 months. She was otherwise healthy and had no history of any drug use. She had no complaints of itching or bleeding on the lesions. There was no freckling on her face and she did not describe photosensitivity. There was no finding of syndromes characterized by multiple lentigines, no known genetic disease in the family, and no intermittent or continuous sunlight exposure for the respective sites. In the dermatological examination, hundreds of dome-shaped nevi with reddish brown colors, <0.5 cm in diameter were observed on the legs and upper body, especially on the buttocks (Fig. 1). A few lesions compatible with Spitz nevus were observed on the neck, which were reported to have newly formed.

**Figure 1:**
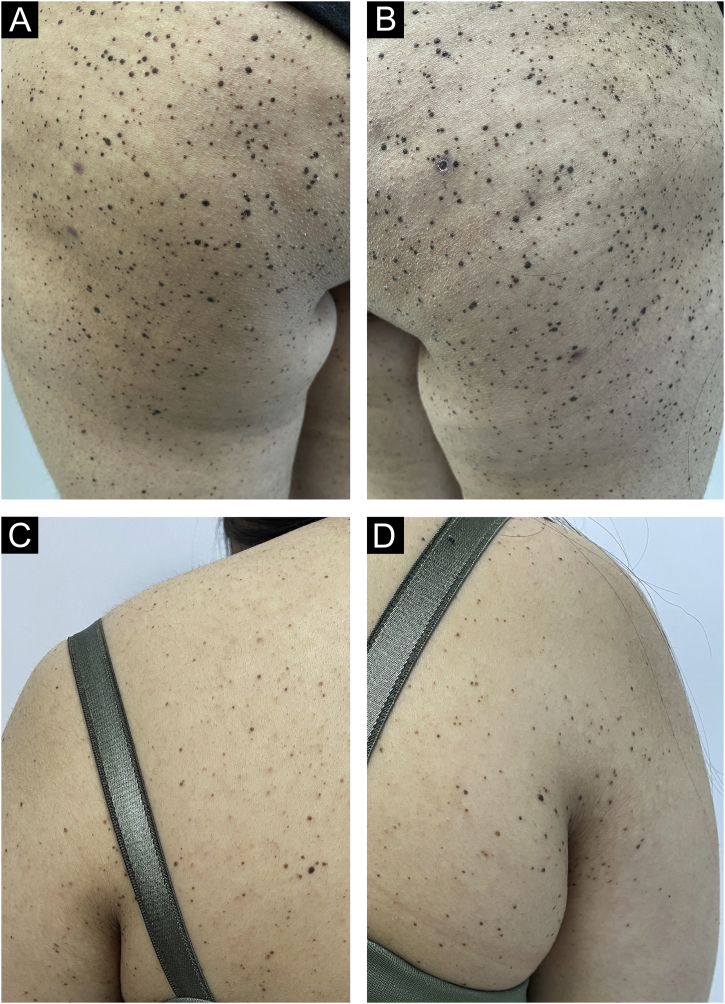
Hundreds of dome-shaped, hyperpigmented nevi on bilateral hips, legs and trunk (A‒C).

On dermatoscopic examination, there is a reticular distribution, which is darker in the center and becomes lighter in the periphery, and globules are observed in the periphery of the lesions (Fig. 2).

**Figure 2:**
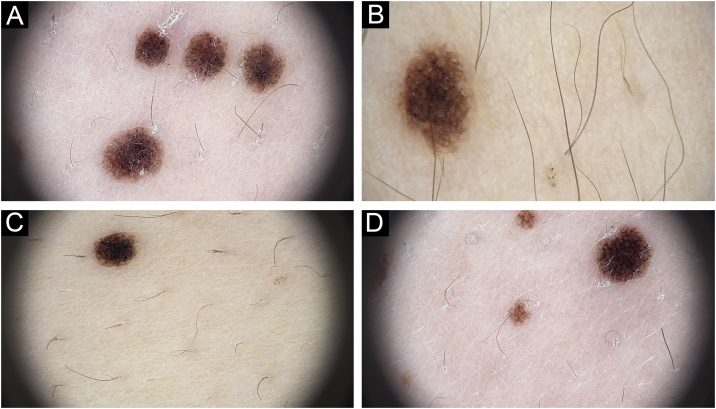
Dermatoscopic images of different lesions showing typical reticular distribution (A‒D).

In the hematoxylin-eosin stained sections from four lesions; symmetric, junctional, localized, pigmented discohesive melanocytic nest structures with spindle cells and pagetoid spreading areas are seen (Fig. 3). No mild atypia was observed in melanocytes. Diffuse and strong positive staining was observed with S100, MelanA, HMB45. In addition, PRAME is negative, with no loss of expression with BAP-1. Thus, metastatic melanoma and Spitz nevi with loss of BAP-1 expression, which come to mind in the differential diagnosis were excluded (Fig. 4).

**Figure 3:**
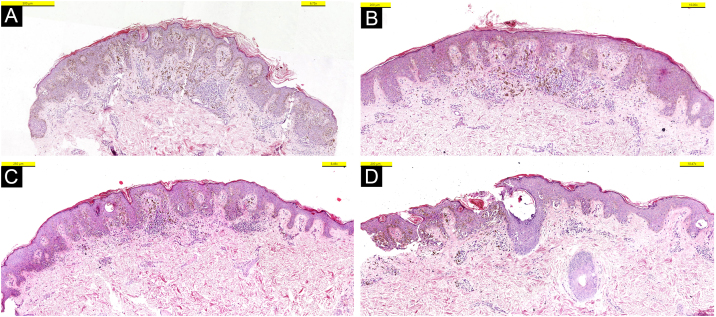
Hematoxylin-eosin stained sections of four different lesions (A‒D); Symmetric junctional Spitz nevus with no mild atypia is seen.

**Figure 4:**
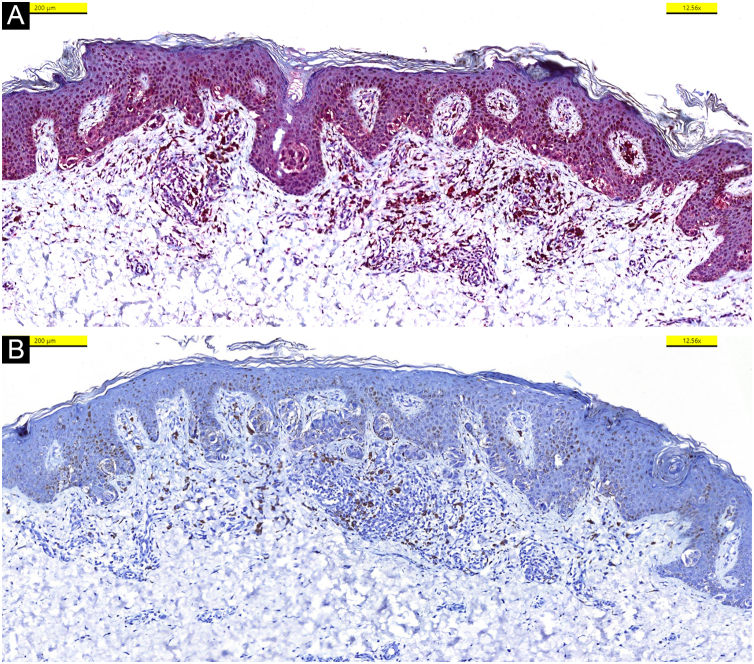
Nuclear expression of BAP-1 in keratinocytes and melanocytes (A), no immunohistochemical staining was observed with PRAME (B).

One lesion larger than the others was excised for genetic analysis. No mutations were detected in the HRAS gene sequence in next-generation sequencing (NextGENe and Geneticist Assistant) and the ROS1 gene in FISH analyses (CytoCell ROS1 Breakapart FISH Probe).

In the light of clinical and pathological data, the patient was diagnosed with eruptive disseminated Spitz nevus. Strong protection from sunlight and follow-up at 6-month intervals was recommended.

Spitz nevi are melanocytic lesions in dome-like morphology which are usually observed in pinkish red color, but may also be in brown tones. Among Spitz nevi, solitary forms are the most common variant. The eruptive form is extremely rare and has been reported mostly in adolescence and later. In the reported cases, the lesions generally formed and spread over months to years. Interestingly, in this case, the lesions spread very rapidly in a short period of 2 months.

Although the exact etiopathogenesis is not known, there are some theories. Some factors such as drugs, the use of cytokines, immunosuppression, inflammatory conditions, pregnancy, atopic dermatitis, and radiotherapy are considered to be responsible in the formation of lesions, it has also been reported in completely healthy people, as in this case.^1^, ^2^, ^3^, ^4^, ^5^, ^6^

To date, some theories regarding the etiopathogenesis have been proposed. One view is that the nevi probably spread along the neurovascular and lymphatic networks. Sentinel lymph node biopsies have been shown as evidence for this view.^7^ Embryonic mosaicism has been suggested because of the cutaneous spread pattern.^7^ According to another view, the tropism of nevus cells to the places where lesions will occur has been suggested, and it has been stated that it waits without proliferating in the skin until a trigger factor occurs.^8^

HRAS, ROS1, NTRK1, ALK, BRAF and RET gene mutations have been shown in many Spitz nevus cases. The fact that these gene mutations are not encountered in all eruptive forms brings to mind that there may be other genetic pathways.^7^, ^9^, ^10^, ^11^ No mutations were detected in the HRAS and ROS1 genes in this eruptive Spitz nevus case.

Table 1 presents the summary of demographic, clinical, and genetic analysis results of eruptive Spitz nevus cases that underwent genetic analysis.

**Table 1:** General characteristics and genetic analysis results of eruptive Spitz nevus cases genetically analyzed to date (While creating this table, the article by Fernandez-Flores et al.^15^ was used and expanded).

	Age/Sex	Number of lesions	Localization	Formation time	Immunohystochemistry and genetic analyses	
Morgan et al.^12^	30/M	Several hundred	Trunk and extremities	Last 2-years	S100 and MIB-1: low mitotic activity	Blood and nevoid fibroblasts karyotype analyse was normal
Boone et al.^13^	17/F	More than 50	Head, neck, arms, thighs, and buttocks	Last 1-year		Tetraploid cells showed balanced gains in 6p25 (RREB1), 6q23 (MYB), 11q13 (cyclin D1), and Cep6, with all cells having 3 or 4 identifiable copies of each chromosomal segment
Boone et al.^13^	51/F	Several	Groins, left buttock, right hip, upper thigh	Last 16-years		No gains or loses of chromosomes 11 and 16
Gantner et al.^11^	16/M	Several	Trunk, face, arms, legs and the penis	Last several months		BRAF, HRAS, KRAS, and NRAS hotspot mutations
						No mutations of the BRAF, RAS and CDKN2A genes
Feito-Rodríguez et al.^14^	Several months/F	Multiple	Extremities	From the tenth day of birth		No mutation was detected in the comparative genomic hybridization analysis
Raghavan et al.^7^	49/F	More than 100	Buttocks, trunk, extremities.	For 4-years after diagnosis of Spitz nevus		TMP3-ROS1 rearrangement with identical intronic breakpoints
						Loss of chromosome 12q
Fernandez-Flores et al.^15^	29/F	Dozens	All over the body	Last 19-years	PRAME-ROS-1-PDL-1- panTRK- ALK-	Next-generation sequencing: No NTRK1, NTRK2, or NTRK3 fusions
						FISH for PTEN showed no alteration
Our case	16/F	Hundreds	Spreading from the hips to the legs and trunk	Last 2-months	HMB45+ S100+ MelanA+ **PRAME−, no loss of expression with BAP-1**	No mutations were detected in the HRAS gene sequence in next generation sequencing and in the ROS1 gene in FISH analyzes.

In conclusion, the etiopathogenesis of eruptive Spitz nevus has not been fully elucidated. Considering the age range of the affected people; the idea that Spitzoid melanocytes proliferate as a result of an as-yet-unknown trigger on the appropriate genetic background seems plausible. The fact that there is a great difference in the speed of formation of nevi in the reported reports may indicate different processes in different people rather than a single pathophysiological process. There is already a wide variety of results in genetic analysis. Much more study is needed to fully understand this phenomenon.

## Financial support

None declared.

## Authors’ contributions

Emre Zekey: Literature searching, designing and writing the manuscript.

Seher Darakcı: Histopathological examination.

## Conflicts of interest

None declared.

## References

[1] Betlloch I., Amador C., Chiner E., Pasquau F., Calpe J.L., Vilar A. (1991). Eruptive melanocytic nevi in human immunodeficiency virus infection. Int J Dermatol.

[2] López V., Molina I., Martín J.M., Santonja N., Forner M.J., Jordá E. (2010). Eruptive nevi in a patient receiving cyclosporine A for psoriasis treatment. Arch Dermatol.

[3] Lanschuetzer C.M., Emberger M., Hametner R., Klausegger A., Pohla-Gubo G., Hintner H. (2003). Pathogenic mechanisms in epidermolysis bullosa naevi. Acta Derm Venereol.

[4] Cardones A.R., Grichnik J.M. (2009). alpha-Melanocyte-stimulating hormone-induced eruptive nevi. Arch Dermatol.

[5] Onsun N., Saraçoğlu S., Demirkesen C., Kural Y.B., Atilganoğlu U. (1999). Eruptive widespread Spitz nevi: can pregnancy be a stimulating factor?. J Am Acad Dermatol.

[6] Fass J., Grimwood R.E., Kraus E., Hyman J. (2002). Adult onset of eruptive widespread Spitz’s nevi. J Am Acad Dermatol.

[7] Raghavan S.S., Kapler E.S., Dinges M.M., Bastian B.C., Yeh I. (2020). Eruptive Spitz nevus, a striking example of benign metastasis. Sci Rep.

[8] Ross A.L., Sanchez M.I., Grichnik J.M. (2011). Nevogenesis: a benign metastatic process?. ISRN Dermatol.

[9] Wiesner T., He J., Yelensky R., Esteve-Puig R., Botton T., Yeh I. (2014). Kinase fusions are frequent in Spitz tumours and spitzoid melanomas. Nat Commun.

[10] Bastian B.C., LeBoit P.E., Pinkel D. (2000). Mutations and copy number increase of HRAS in Spitz nevi with distinctive histopathological features. Am J Pathol.

[11] Gantner S., Wiesner T., Cerroni L., Lurkin I., Zwarthoff E.C., Landthaler M. (2011). Absence of BRAF and HRAS mutations in eruptive Spitz naevi. Br J Dermatol.

[12] Morgan C.J., Nyak N., Cooper A., Pees B., Friedmann P.S. (2006). Multiple Spitz naevi: a report of both variants with clinical and histopathological correlation. Clin Exp Dermatol.

[13] Boone S.L., Busam K.J., Marghoob A.A., Fang Y., Guitart J., Martini M. (2011). Two cases of multiple spitz nevi: correlating clinical, histologic, and fluorescence in situ hybridization findings. Arch Dermatol.

[14] Feito-Rodríguez M., Lucas-Laguna R., Bastian B.C., Leboit P., Gónzalez-Beato M.J., López-Gutiérrez J.C. (2011). Nodular lesions arising in a large congenital melanocytic naevus in a newborn with eruptive disseminated Spitz naevi. Br J Dermatol.

[15] Fernandez-Flores A., Cassarino D. (2022). Genetic studies on a case of eruptive disseminated spitz nevus and review of other 33 cases. Am J Dermatopathol..

